# 1603. Efficacy, safety and tolerability of switching to dolutegravir/lamivudine in virologically suppressed adults living with HIV on bictegravir/emtricitabine/tenofovir alafenamide -the DYAD study

**DOI:** 10.1093/ofid/ofad500.1438

**Published:** 2023-11-27

**Authors:** Charlotte-Paige M Rolle, Jamie Castano, Vu Nguyen, Federico Hinestrosa, Edwin DeJesus

**Affiliations:** Orlando Immunology Center, Orlando, FL, USA, Orlando, Florida; Orlando Immunology Center, Orlando, Florida; Orlando Immunology Center, Orlando, Florida; Orlando Immunology Center, University of Central Florida College of Medicine, Orlando, FL; Orlando Immunology Center, University of Central Florida College of Medicine, Orlando, FL

## Abstract

**Background:**

In TANGO, switching to dolutegravir/lamivudine (DTG/3TC) was noninferior compared to continuing a tenofovir alafenamide (TAF)-based regimen, however switching from bictegravir (BIC)/emtricitabine (F)/TAF was not evaluated. Here, we present efficacy and safety of switching to DTG/3TC compared with continuing B/F/TAF in virologically suppressed adults through 24 weeks.

**Methods:**

DYAD (NCT04585737) is an ongoing, open-label clinical trial that randomized adults with HIV-1 RNA< 50 copies/mL and no prior virologic failure (2:1) to switch to once-daily fixed-dose DTG/3TC or remain on B/F/TAF. The primary endpoint is the proportion with HIV-1 RNA ≥ 50 c/mL at Week (W) 48 (FDA snapshot algorithm, ITT-E population). A planned W24 interim analysis assessed noninferiority with a 6% margin.

**Results:**

Overall, 222 adults (16% women; 51% aged ≥50 years; 28% Black) were randomized. At W24, 3 (2%) participants on DTG/3TC and 3 (4%) on B/F/TAF had HIV-1 RNA ≥ 50 c/mL (adjusted treatment difference -2.1%, 95% confidence interval [-9.5%, 2.5%]) meeting noninferiority criteria. Through W24, 4 on DTG/3TC and 3 on B/F/TAF met confirmed virologic withdrawal (CVW) criteria and underwent resistance testing. 2/7 had treatment-emergent resistance. One B/F/TAF CVW developed M184M/I and G140G/S at W12; at study discontinuation (DC), HIV-1 RNA< 50 c/mL and the participant remained on B/F/TAF. One DTG/3TC CVW had no resistance on study genotype but underwent repeat genotypic testing outside the study (ordered by clinic provider) and had M184V at W12; at study DC, HIV-1 RNA< 50 c/mL on DTG/3TC and the participant was subsequently transitioned to DTG + darunavir/cobicistat (DRV/c). One non-CVW DTG/3TC participant developed M184V and K65R at W12 (genotype inadvertently collected at first episode of unconfirmed viremia); at study DC, HIV-1 RNA< 50 c/mL on DTG/3TC and the participant was subsequently transitioned to DTG+DRV/c. Drug-related adverse events (AEs) and withdrawals due to AEs occurred in 31 (21%) and 6 (4%) participants with DTG/3TC and 1 (1%) and 0 participants with B/F/TAF, respectively.

**Figure:**
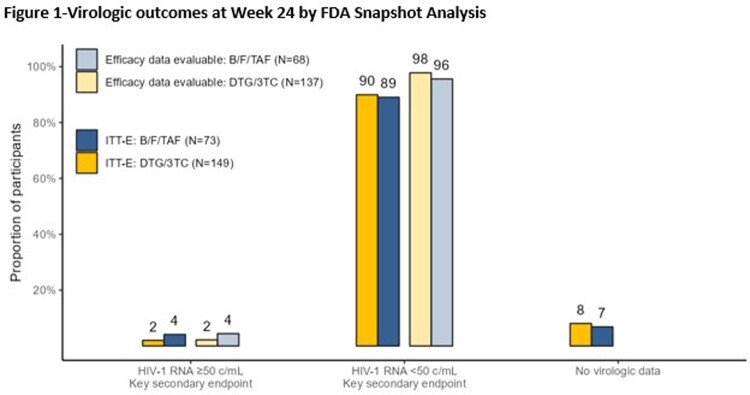

**Figure:**
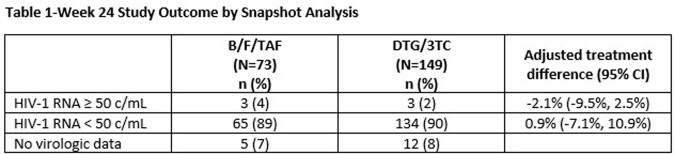

**Conclusion:**

DYAD demonstrated noninferior efficacy of switching to DTG/3TC vs. continuing B/F/TAF at W24. Higher AE rates in the DTG/3TC arm are likely consistent with the open-label nature of this switch study.

**Disclosures:**

**Charlotte-Paige M. Rolle, MD, MPH**, Gilead: Advisor/Consultant|Gilead: Grant/Research Support|Gilead: Honoraria|Janssen: Advisor/Consultant|ViiV: Advisor/Consultant|ViiV: Grant/Research Support|ViiV: Honoraria **Federico Hinestrosa, MD**, AbbVie: Honoraria|Gilead Sciences: Advisor/Consultant|Gilead Sciences: Honoraria|MSD: Honoraria|ViiV Healthcare: Advisor/Consultant|ViiV Healthcare: Honoraria **Edwin DeJesus, MD**, Gilead Sciences, Inc: Advisor/Consultant|Theratechnology: Advisor/Consultant|Theratechnology: Honoraria

